# Updating the diagnosis and management of elevated serum ferritin levels in the era of routine ferritin testing of blood donors by Australian Red Cross Lifeblood

**DOI:** 10.5694/mja2.52618

**Published:** 2025-02-27

**Authors:** Gary D Zhang, James Chen, Daniel M Johnstone, Martin B Delatycki, Katie Allen, John K Olynyk

**Affiliations:** ^1^ Fiona Stanley Hospital Perth WA; ^2^ The University of Western Australia Perth WA; ^3^ University of Newcastle Newcastle NSW; ^4^ Victorian Clinical Genetics Services Royal Children's Hospital Melbourne VIC; ^5^ Bruce Lefroy Centre Murdoch Children's Research Institute Melbourne VIC; ^6^ University of Melbourne Melbourne VIC; ^7^ Murdoch Children's Research Institute Melbourne VIC; ^8^ Curtin University Perth WA

**Keywords:** Hemochromatosis, Liver diseases, Blood banks

Elevated serum ferritin levels (hyperferritinaemia) can reflect a wide range of conditions and are not solely indicative of iron overload. A thorough clinical assessment is required to identify serious causes of high serum ferritin levels, evaluate iron status and manage associated complications.

The upper limit of the reference interval for serum ferritin values varies between laboratories due to different testing methods, and the Royal College of Pathologists of Australasia no longer specifies upper limits in its guidance to laboratories.^1^ Up to 20% of men have elevated serum ferritin concentrations, while the prevalence in women varies from 3% in premenopausal women to 17% in women older than 70 years.^2^ Less than 10% of cases of hyperferritinaemia are due to iron overload.^3^, ^4^, ^5^

In August 2023, the Australian Red Cross Lifeblood introduced routine ferritin testing for its new blood donors (over 100 000 people per year),^6^ with plans to expand testing to all whole blood donors. Donors are formally advised if their results are outside the sex‐based reference intervals of 15–400 μg/L for female donors and 30–500 μg/L for male donors.^7^ Based on Australian adult population data, about 10% of men and 2% of women will exceed these upper limit thresholds of serum ferritin levels.^8^ This will likely result in a considerable increase in adults with elevated serum ferritin levels being identified and referred to their primary care physician for evaluation. This medical education article provides a guide for the investigation and management of elevated serum ferritin levels.

## Diagnosis

Serum ferritin levels may be elevated due to either an iron overload disorder or a non‐iron overload disorder, with these aetiologies described in Box 1. The key principle guiding assessment of elevated serum ferritin values in adults is to differentiate between iron overload and non‐iron overload causes, as this guides decision making related to treatment. Non‐iron overload hyperferritinaemia occurs from the acute phase reactant properties of ferritin, and is commonly observed in inflammation, obesity, infections, malignancy and hepatocellular injury.^5^

Box 1 **Aetiologies of persistent hyperferritinaemia, stratified by the presence or absence of iron overload^5^, ^10^, ^11^**

**Table:** 

Without iron overload	With iron overload
Infections	Hereditary haemochromatosis (*HFE* haemochromatosis and non‐*HFE* haemochromatosis)
Acute	
Chronic	
Metabolic syndrome	Iatrogenic
	Excess iron supplementation (oral and/or intravenous)
	Frequent/excess blood transfusions
Alcohol excess	Haemoglobinopathies/iron loading anaemias
Liver disease (metabolic dysfunction‐associated steatotic liver disease, viral hepatitis, other)	Porphyria cutanea tarda
Inflammatory conditions	Other genetic causes (aceruloplasminaemia)
Rheumatological and immunological conditions	
Malignancy	
Thyrotoxicosis

The approach to investigating an elevated serum ferritin result is described in Box 2. Individuals may have existing diagnoses that increase serum ferritin levels, such as inflammatory conditions, chronic liver disease, malignancy, excessive iron supplementation, or recurrent blood transfusions.^5^ The initial clinical assessment should consider the following more common non‐iron overload causes: acute illness, infections (full blood count and C‐reactive protein tests), alcohol intake, risk factors for liver disease (liver function tests and abdominal ultrasonography), metabolic syndrome or dysfunction (lipid studies and fasting serum glucose or glycated haemoglobin test^9^), family history (iron overload and other associated conditions), as red flags for malignancy, chronic inflammatory conditions and renal impairment.^5^, ^10^, ^11^

Box 2 **A suggested approach to the assessment of individuals with elevated serum ferritin levels**

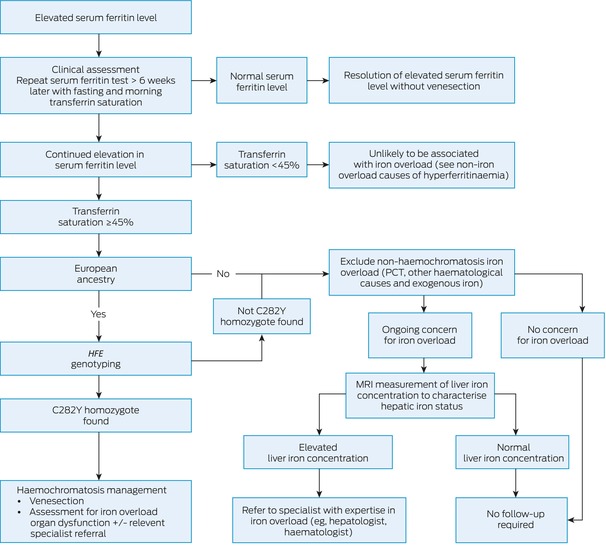

C282Y = p.Cys282Tyr; MRI = magnetic resonance imaging; PCT = porphyria cutanea tarda.

A follow‐up measurement of the serum ferritin level should be repeated at least six weeks after an elevated result to allow for sufficient time for acute illness to resolve, along with measurement of the serum transferrin saturation level in the morning and while fasted to reduce the variabilities in serum iron levels due to diet and diurnal fluctuations.^12^ A serum transferrin saturation value of 45% or greater is consistent with, but not diagnostic of, iron overload, and predicts *HFE*‐related haemochromatosis with high sensitivity in people with European ancestry, with care taken in determining this aspect in multicultural societies such as Australia.^13^ Iron overload is unlikely if the transferrin saturation value is less than 45%.^3^, ^13^, ^14^

As shown in Box 2, if the repeat testing demonstrates both the serum ferritin level and transferrin saturation within the reference interval, no further action is required, as treatable iron overload disorders are highly unlikely.^11^, ^13^, ^14^ If the serum ferritin level and transferrin saturation are both elevated, further assessment should be undertaken for the presence of *HFE*‐related haemochromatosis, due to p.Cys282Tyr homozygosity, in individuals with European ancestry or if there is uncertainty regarding European ancestry. Likewise, testing for the *HFE* p.Cys282Tyr mutation is strongly suggested when there is a family history of haemochromatosis or iron overload,^4^, ^13^ as p.Cys282Tyr homozygosity accounts for almost all *HFE* haemochromatosis in Australia, with the remaining genotypic variations of little or no clinical significance.^13^, ^14^, ^15^, ^16^ Further diagnosis and management strategies for haemochromatosis are covered elsewhere.^13^, ^14^ If *HFE* haemochromatosis is excluded, further assessment for the presence of porphyria cutanea tarda (PCT) or other haematological causes of iron overload is indicated. Where there is doubt as to the presence or absence of iron overload, quantification of liver iron concentration (LIC) using an available magnetic resonance imaging (MRI) method is indicated.^17^, ^18^

Specialist haematology or hepatology referral for patients with a serum ferritin value greater than 1000 μg/L but no identified iron overload disorder should only be considered when elevation of the LIC above the reference interval has been confirmed with an available MRI‐based method.^13^ A LIC within the reference interval excludes iron overload as a cause of hyperferritinaemia and precludes the need for iron‐focused management of the elevated serum ferritin level.

Treatment targeting iron overload as the cause of elevated ferritin levels is only clinically indicated for *HFE*‐related haemochromatosis, PCT, or secondary iron overload associated with exogenous iron administration or haematological disorders, such as thalassaemia and other haemoglobinopathies. Therapeutic phlebotomy is the treatment of choice only for hyperferritinaemia caused by *HFE*‐related haemochromatosis or PCT. There is no indication for any iron reduction treatment when a non‐iron overload disorder has been diagnosed as the cause of hyperferritinaemia.^13^ Further management and complications of *HFE* haemochromatosis and other iron overload disorders are discussed in detail elsewhere.^10^, ^13^, ^14^, ^19^

## Conclusion

Serum ferritin levels above the reference interval must always be assessed further. Iron overload accounts for less than 10% of individuals with hyperferritinaemia, and when present is usually associated with an elevated serum transferrin saturation above 45%. In individuals with European ancestry, *HFE* genotyping must be performed when persistent elevation of serum ferritin levels and transferrin saturation are confirmed, or when there is a family history of the disorder. Hyperferritinaemia without iron overload can result from a wide range of potentially serious causes, which should be evaluated initially with routine investigations, but these do not require specific treatment to reduce body iron or serum ferritin levels as there is no evidence for any benefit to be derived from such interventions.

## Competing interests

No relevant disclosures.

## Provenance

Not commissioned; externally peer reviewed.
